# Supplementary material for: Assessment of the Impact of COVID-19 Lockdown on the Nutritional Status and Lipid Profile of Employees in a Teaching Hospital in Rome: A Retrospective Cohort Study
Source: Int J Environ Res Public Health. 2022 Apr 9;19(8):4549. doi: 10.3390/ijerph19084549 (PMC9027901; doi:10.3390/ijerph19084549)
Supplement: Supplementary file 1 [file ijerph-19-04549-s001.zip › ijerph-1632675-supplementary.pdf]

Table S1. Linear regression model of Δ BMI

Variables	Standardized Beta (p) *Full model*	Standardized Beta (p) *Stepwise*	Standardized Beta (p) *Females*	Standardized Beta (p) *Males*	Standardized Beta (p) *Over 52 years*	Standardized Beta (p) *Under 52 years*	Standardized Beta (p) *Subgroup with PA*
**Gender**							
Males*							
Females	-0,018 (0,603)	-	-	-	-	-	-0,131 (**0,006**)
**Age**	-0,064 (0,065)	-0,058 (0,069)	-	-0,127 (**0,017**)	-0,107 (**0,014**)	-	-0,080 (0,098)
**Role**							
Physicians	-0,032 (0,702)	-	-0,094 (**0,018**)	-	-	-	-
Nurses	0,063 (0,476)	0,119 (**<0,001**)		0,123 (**0,018**)	-	0,130 (**0,004**)	0,166 (**0,001**)
Technicians	-0,027 (0,689)	-	-	-	-	-	-
Administratives*							
**Judgment**							
Eligible*							
With Limitation	-0,027 (0,412)	-	-	-	-	-	-
**Biological**							
No*							
Yes	-0,050 (0,112)	-	-0,108 (**0,009**)	-	-	-	-
**VDU**							
No*							
Yes	0,049 (0,164)	0,060 (0,064)	-	-	-	-	0,084 (0,076)
**MHL/MPH**							
No*							
Yes	0,029 (0,538)	-	-	-	-	-	-
**Night shift**							
No*							
Yes	-0,040 (0,281)	-	-	-0,122 (**0,019**)	-	-	-
**Chemical**							
No*							
Yes	-0,025 (0,449)	-	-0,102 (**0,014**)	-	-	-	-
**Smoker**							
No*							
Yes	0,043 (0,178)	-	-	-	-	0,096 (**0,037**)	-
**Diabetes**							
No*							
Yes	-0,024 (0,457)	-	-	-	-	-	-
**Hypertension**							
No*							
Yes	0,011 (0,734)	-	-	-	-	-	-
**Heart Disease**							
No*							
Yes	0,073 (**0,023**)	0,070 (**0,025**)	-	0,092 (0,067)	0,104 (**0,017**)	-	-

**Thyroid Disease**							
No*							
Yes	0,050 (0,117)	-	-0,069 (0,083)	-	0,080 (0,067)	-	-
**Cancer**							
No*							
Yes	-0,020 (0,523)	-	-	-	-	-	-
**AUDIT-C**	-0,029 (0,379)	-	-0,089 (**0,026**)	-	-	-0,106 (**0,022**)	-
**Physical Activity**							
No*							
Yes	-	-	-	-	-	-	-0,114 (**0,015**)
**R^2**	0,034	0,023	0,04	0,048	0,028	0,04	0,056

*reference group

Table S2. Linear regression model of Δ LDL

Variables	Standardized Beta (p) *Full model*	Standardized Beta (p) *Stepwise*	Standardized Beta (p) *Females*	Standardized Beta (p) *Males*	Standardized Beta (p) *Over 52 years*	Standardized Beta (p) *Under 52 years*	Standardized Beta (p) *Subgroup with PA*
**Gender**							
Males*							
Females	0,034 (0,325)	-	-	-	-	-	-
**Age**	0,030 (0,388)	-	-	-	-	-	-
**Role**							
Physicians	0,117 (0,160)	0,078 (**0,013**)	-	0,094 (0,058)	0,092 (**0,034**)	-	0,180 (**<0,001**)
Nurses	0,019 (0,827)	-	-0,080 (**0,048**)	-	-	-0,083 (0,093)	-
Technicians	0,017 (0,804)	-	-	-	-	-0,112 (**0,026**)	-
Administratives*							
**Judgment**							
Eligible*							
With Limitation	-0,016 (0,632)	-	-	-	-	-	-
**Biological**							
No*							
Yes	-0,008 (0,797)	-	-	-	-	-	-
**VDU**							
No*							
Yes	-0,010 (0,766)	-	-	-	-	-	-0,079 (0,076)
**MHL/MPH**							
No*							
Yes	0,038 (0,408)	-	-	-	-	-	-
**Night shift**							
No*							
Yes	-0,023 (0,533)	-	-	-	-	-0,123 (**0,008**)	-
**Chemical**							
No*							
Yes	-0,059 (0,071)	-0,062 (**0,047**)	-0,099 (**0,015**)	-	-0,079 (0,068)	-	-
**Smoker**							
No*							
Yes	0,018 (0,572)	-	-	-	-	-	-
**Diabetes**							
No*							
Yes	-0,053 (0,095)	-	-	-	-0,098 (**0,025**)	-	-0,096 (**0,030**)
**Hypertension**							
No*							
Yes	0,035 (0,291)	-	-	-	-	-	-
**Heart Disease**							
No*							
Yes	-0,119 (**<0,001**)	-0,123(**<0,001**)	-	-0,196 (**<0,001**)	-0,137 (**0,002**)	-	-0,140 (**0,002**)

**Thyroid Disease**							
No*							
Yes	-0,053 (0,099)	-	-0,085 (**0,034**)	-	-	-	-0,086 (**0,051**)
**Cancer**							
No*							
Yes	-0,057 (0,072)	-0,055 (0,076)	-	-0,092 (0,062)	-	-0,088 (0,052)	-0,192 (**<0,001**)
**AUDIT-C**	0,004 (0,909)	-	-	-	-	-	-
**Physical Activity**							
No*							
Yes							-0,086 (**0,058**)
**R^2**	0,035	0,026	0,022	0,054	0,044	0,027	0,107

*reference group

Table S3. Linear regression model of Δ total cholesterol

Variables	Standardized Beta (p) *Full model*	Standardized Beta (p) *Stepwise*	Standardized Beta (p) *Females*	Standardized Beta (p) *Males*	Standardized Beta (p) *Over 52 years*	Standardized Beta (p) *Under 52 years*	Standardized Beta (p) *Subgroup with PA*
**Gender**							
Males*							
Females	0,044 (0,213)	-	-	-	-	-	-
**Age**	0,050 (0,147)	-	0,083 (**0,044**)	-	-	-	-
**Role**							
Physicians							
Nurses	0,104 (0,210)	0,056 (0,071)	-	-	-	-0,272 (0,052)	0,146 (**0,001**)
Technicians	-0,002 (0,980)		-0,153 (**0,003**)		-	-0,355 (**0,018**)	-
Administratives*	0,041 (0,539)	-	-	-	-	-0,267 (**0,007**)	-
**Judgment**							
Eligible*							
With Limitation	0,003 (0,935)	-	-	-	-	-	-
**Biological**							
No*							
Yes	-0,027 (0,386)	-	-	-	-	-	-
**VDU**							
No*							
Yes	-0,030 (0,393)	-	-	-	-	-	-
**MHL/MPH**							
No*							
Yes	0,061 (0,187)	-	0,098 (0,055)	-	-	-	-
**Night shift**							
No*							
Yes	-0,038 (0,300)	-	-	-	-	-0,115 (**0,013**)	-
**Chemical**							
No*							
Yes	-0,057 (0,079)	-	-0,095 (**0,019**)	-	-	-	-
**Smoker**							
No*							
Yes	0,005 (0,886)	-	-	-	-	-	-
**Diabetes**							
No*							
Yes	-0,036 (0,263)	-	-	-	-0,082 (0,062)	-	-
**Hypertension**							
No*							
Yes	0,025 (0,444)	-	-	-	-	-	-
**Heart Disease**							
No*							
Yes	-0,138 (**<0,001**)	-0,133 (**<0,001**)	-	-0,207(**<0,001**)	-0,155 (**<0,001**)	-	-0,148 (**0,001**)
**Thyroid Disease**							
No*							
Yes	-0,040 (0,205)	-	-0,071 (0,077)	-	-	-	-
**Cancer**							
No*							
Yes	-0,051 (0,106)	-	-	-0,110 (**0,025**)	-	-0,176(**<0,001**)	-0,194 (**<0,001**)
**AUDIT-C**	0,006 (0,853)	-	-	-	-	-	-

**Physical Activity**							
No*							
Yes							-0,097 (**0,033**)
**R^2**	0,035	0,02	0,032	0,054	0,035	0,05	0,075

*reference group
